# Genomic Characterization of Methicillin-Resistant and Methicillin-Susceptible *Staphylococcus aureus* Implicated in Bloodstream Infections, KwaZulu-Natal, South Africa: A Pilot Study

**DOI:** 10.3390/antibiotics13090796

**Published:** 2024-08-23

**Authors:** Bakoena A. Hetsa, Jonathan Asante, Joshua Mbanga, Arshad Ismail, Akebe L. K. Abia, Daniel G. Amoako, Sabiha Y. Essack

**Affiliations:** 1 Antimicrobial Research Unit, College of Health Sciences, University of KwaZulu-Natal, Durban 4000, South Africa; jonathan.asante@ucc.edu.gh (J.A.); mbangaj@ukzn.ac.za (J.M.); abiaakebel@ukzn.ac.za (A.L.K.A.); amoakod@ukzn.ac.za (D.G.A.); essacks@ukzn.ac.za (S.Y.E.); 2 School of Pharmacy and Pharmaceutical Sciences, University of Cape Coast, PMB, Cape Coast, Ghana; 3 Department of Applied Biology & Biochemistry, National University of Science and Technology, Corner Cecil Avenue & Gwanda Road, Bulawayo 263, Zimbabwe; 4 Sequencing Core Facility, National Institute for Communicable Diseases, Division of the National Health Laboratory Service, Johannesburg 2193, South Africa; arshadi@nicd.ac.za; 5 Department of Biochemistry and Microbiology, Faculty of Science, Engineering and Agriculture, University of Venda, Thohoyandou 0950, South Africa; 6 Department of Pathobiology, University of Guelph, Guelph, ON N1G 2W1, Canada; 7 School of Pharmacy, University of Jordan, Amman 11942, Jordan

**Keywords:** *Staphylococcus aureus*, bloodstream infections, whole-genome sequencing, antibiotic resistance, virulence, bioinformatics

## Abstract

*Staphylococcus aureus* is an opportunistic pathogen and a leading cause of bloodstream infections, with its capacity to acquire antibiotic resistance genes posing significant treatment challenges. This pilot study characterizes the genomic profiles of *S. aureus* isolates from patients with bloodstream infections in KwaZulu-Natal, South Africa, to gain insights into their resistance mechanisms, virulence factors, and clonal and phylogenetic relationships. Six multidrug-resistant (MDR) *S. aureus* isolates, comprising three methicillin-resistant *S. aureus* (MRSA) and three methicillin-susceptible *S. aureus* (MSSA), underwent whole genome sequencing and bioinformatics analysis. These isolates carried a range of resistance genes, including *blaZ*, *aac(6′)-aph(2″)*, *ant(9)-Ia*, *ant(6)-Ia*, and *fosB*. The *mecA* gene, which confers methicillin resistance, was detected only in MRSA strains. The isolates exhibited six distinct *spa* types (t9475, t355, t045, t1265, t1257, and t7888) and varied in virulence gene profiles. Panton–Valentine leukocidin (Luk-PV) was found in one MSSA isolate. Two SCC*mec* types, IVd(2B) and I(1B), were identified, and the isolates were classified into four multilocus sequence types (MLSTs), with ST5 (n = 3) being the most common. These sequence types clustered into two clonal complexes, CC5 and CC8. Notably, two MRSA clones were identified: ST5-CC5-t045-SCC*mec*_I(1B) and the human-associated endemic clone ST612-CC8-t1257-SCC*mec*_IVd(2B). Phylogenomic analysis revealed clustering by MLST, indicating strong genetic relationships within clonal complexes. These findings highlight the value of genomic surveillance in guiding targeted interventions to reduce treatment failures and mortality.

## 1. Introduction

*Staphylococcus aureus* is a Gram-positive bacterium inhabiting healthy individuals’ nostrils and skin. However, it has become an important opportunistic pathogen in communities and hospitals [1]. It causes severe skin infections, pneumonia, endocarditis, and bloodstream infections (BSIs) [2]. BSIs caused by *S. aureus* infections have high morbidity and mortality if not treated timeously [3]. The most significant risk factors for *S. aureus* BSIs are intravascular devices, surgical procedures, and a debilitated immune system [4].

Methicillin-resistant *S. aureus* (MRSA) has become a significant cause of BSIs. MRSA poses a major public health threat because of multidrug resistance to different antibiotic classes that limit treatment options [5]. Methicillin resistance in MRSA strains is mediated by the *mecA* gene, found on a mobile genetic element (MGE) known as the staphylococcal cassette chromosome *mec* (SCC*mec*) [6]. Methicillin-susceptible *S. aureus* (MSSA) is also emerging as a causative agent of BSIs [7] and has been reported to display high virulence and multidrug resistance [8]. 

The pathogenicity of *S. aureus* depends on its ability to produce a wide array of virulence factors involved in adhesion, invasion of host tissues, immune system evasion, and biofilm formation [9,10]. Also, *S. aureus* produces metallophores that enable bacteria to scavenge metal ions such as iron and zinc essential for bacterial metabolism and pathogenicity [11]. Virulence factors and multiple resistance genes can be transmitted by horizontal gene transfer (HGT) [12] on diverse MGEs, amongst which plasmids are reported as the primary sources for dissemination [4]. 

The epidemiology of *S. aureus* strains indicates that its molecular characteristics continually change over time, resulting in new clones, which vary by region. In a study in the United States, ST5 and ST8 were the most prevalent sequence types [13]. In South Africa, ST612 is dominant in the hospital environment [14]. The ST612-IV [2B], belonging to *spa* type t1257, was identified as a typical clone in clinical settings [15] and sporadically in poultry settings [16]. The ST5 and ST8 clones are commonly associated with BSIs and the pandemic lineages of *S. aureus*, such as the clonal complex CC8 and CC5 [17]. Notably, the sequence types ST612, ST5, ST8, and ST72 have displayed high resistance to most antibiotic drug classes and are challenging to treat [17]. 

Multidrug-resistant (MDR) *S. aureus* infections pose a serious clinical concern. A high incidence of pathogenic MDR MRSA has been reported, and the data suggest that its prevalence is increasing in Africa [18]. A recent South African study investigating the genetic relatedness of hospital-acquired-associated MRSA isolates in two hospitals revealed that all isolates were resistant to aminoglycosides and β-lactams. All the isolates carried the *aacA-aphD* and *mecA-*resistant genes and clusters of virulence genes [19]. This pilot study aimed to comprehensively characterize the genomic profiles, resistance mechanisms, virulence factors, pathogenicity, phylogenomic relationships, and clonal diversity of *Staphylococcus aureus* clinical strains implicated in BSIs at a regional hospital in KwaZulu-Natal, South Africa. 

## 2. Results

### 2.1. Patient Demographics and Characteristics

The 6 isolates investigated in this study were obtained from patients who visited a regional hospital in the uMgungundlovu District in the KwaZulu-Natal Province. Three of the six isolates were recovered from the neonatal ICU (n = 3, 50%), two from surgical wards, and one isolate from the pediatric ward. Four patients were male, while two were female. The age distribution of patients ranged from 0 to 33 years old, and the mean age was 8.83 years (Table 1). The demographic details of the source participants of the isolates that were selected for WGS are shown in Supplementary Table S1.

### 2.2. Antibiotic Susceptibility Test Results

The isolates displayed varying phenotypic resistance profiles, with most being resistant to penicillin G (n = 6), tetracycline (n = 5), doxycycline (n = 5), clindamycin (n = 5), moxifloxacin (n = 5), rifampicin (n = 4), and erythromycin (n = 3). The lowest resistance was against nitrofurantoin, tigecycline, and chloramphenicol (n = 1) (Table 1).

### 2.3. Phenotypic and Genotypic Identification of MRSA Isolates

MRSA isolates were confirmed by phenotypic resistance to cefoxitin (Table 1) and the detection of the *mecA* gene using polymerase chain reaction (PCR).

### 2.4. Genomic Features

The genome size of our draft genomes ranged from 2.7 Mb to 2.9 Mb. The genomic characteristics of the sequences in relation to G + C content (%), number of RNAs, number of coding sequences, size, N50, L50, and coverage are shown in Table S2.

### 2.5. In Silico ARGs Analysis

Isolates harbored various permutations and combinations of ARGs, which included ARGs against β-lactams [*blaZ*], aminoglycosides [*aac(6′)-aph(2″)*, *aad(6’)*, *ant(9)-la*, *ant(6)-Ia*, *aph(2″)-Ia*, *aph(3′)-IIa*, *sat-4*], trimethoprim [*dfrG*, *dfrC*], macrolides [*erm(C)*, *erm(A)*], tetracycline [*tet(K)*, *tet(M)*, *mepR*, *mepA*], flouroquinolones [*parE*, *parC*, *grlA*, *gyrA*, *norA*, *norC* (multidrug efflux pumps)], rifampicin [*rpoB*] and fosfomycin [*fosB*, *murA*] (Table 2). Only the MRSA isolates harbored the *mecA* gene. There was good concordance between ARGs and phenotypic profiles for ARGs in all MRSA and MSSA isolates.

#### 2.5.1. MLST, spa Typing, and Clonal Complex

MLST revealed total four sequence types, i.e., ST5 (CC5, n = 3), ST152 (n = 1), ST612 (CC8, n = 1), and ST8 (CC8, n = 1). Two MRSA strains belonged to CC8 and one to CC5. Methicillin-susceptible (MSSA) isolates were identified as ST5 (n = 2) or ST152 (n = 1). The genetic diversity of the isolates was confirmed by *spa* typing, which revealed six different *spa* types: t9475, t1265, t355, t045, t1257, and t7888 (Table 2). CC and *spa* type combinations were CC8-t9475, CC8-t1257, and CC5-t045 among MRSA isolates, with CC5-t1265 belonging to one MSSA isolate. There was no association observed between STs, *spa* type, and CC. The grouping of the STs and *spa*-types yielded six genotypes, i.e., ST8-t9475, ST152-t355, ST5-t045, ST5-t1265, ST612-1257, and ST5-t7888, indicating that isolates were not clonally related.

The SCC*mec*Finder analysis identified two SCC*mec* types, i.e., IVd (2B) and I (1B), among the MRSA isolates (Table 2). One MRSA isolate was non-typeable (NT) for SCC*mec*. The combination of MLST, CC, *spa*, and SCC*mec* yielded the ST612-CC8-t1257-SCC*mec*_IVd (2B) and ST5-CC5-t045-SCCmec_I (1B), clones both of which have been reported in South Africa.

#### 2.5.2. Mobilome (Plasmids, Insertion Sequences, Intact Prophages, and SCCmec Elements)

Analysis of the six isolate genomes identified various MGEs, including plasmid replicons, IS’s, prophages, and SCC*mec* elements. A total of eight different plasmid replicons were detected, of which rep20 (n = 3) was the most prevalent (Table 2). There were no associations between plasmid replicons and STs. However, the rep7c was found in CC8 isolates in addition to other plasmid replicons, while rep16 and rep5a were found in isolates with the non-typeable CC. The rep20 plasmid replicon was associated with CC5 and CC8 isolates. The rep10 was carried in CC8 and CC5 isolates, while the re7a and rep21 were carried in CC8 and CC5 isolates, respectively. IS6 and IS256 were identified in three isolates, and their occurrence was not associated with any STs or CC (Table 2). The ddistribution of ISs and plasmid replicons detected among the isolates are shown in Supplementary Table S4. A total of six intact prophages were detected, of which the most identified were PHAGE_Staphy_phi2958PVL (n = 2) and PHAGE_Staphy_P282 (n = 2) (Table S5). PHAGE_Staphy_phiJB was associated with the *dfrG* gene.

#### 2.5.3. Virulome and Pathogenicity of S. aureus Strains

A total of 82 virulence genes were detected across the isolates (Table S3). The virulence genes belonged to the five main virulence determinant classes of *S. aureus*: adherence factors, immune evasion, enzymes (exoenzymes), toxins, and the secretion system. It is noteworthy that the most prevalent toxins were hemolysins, i.e., gamma (*hlg*), delta (*hld*), alpha (*hly/hla*), staphylococcal enterotoxins (*se*, *set*, *sel*) genes, and leucocidin genes (*lukD/E*), while *lukS-PV* and *lukF-PV* genes were detected in two isolates (S24 and S29). The prediction of isolates pathogenicity towards humans yielded a high average probability score (Pscore ≈ 0.980).

### 2.6. Genetic Environment of the ARGs and Virulence Genes

The co-carriage of ARGs and virulence genes was evident across the isolates. Using NCBI annotation, we identified *blaZ* genes on five isolates in parallel with *cacD*, virulence genes, and the type 1 toxin–antitoxin system. Across the isolates, most *blaZ* genes were associated with regulator genes *blaR* and *blaI* and frequently found with either a recombinase, integrase, cadmium resistance (*cadD*) gene, or type I toxin–antitoxin system (Table 3). A similar genetic context was detected in the S13 isolate, where *blaZ*, *blaR*, and *blaI* were flanked by IS6, *cadD*, a type I toxin–antitoxin system, on a contig with the closest nucleotide homology to a plasmid from *S. aureus* pER10678.3A.1 (CP051928.1), suggesting that ARGs, heavy metal resistance genes (HMRGs), and virulence genes may be mobilized by plasmids (Table 3). It is noteworthy that IS1182 was associated with the *mecA*, *mecI*, and *mecR1* genes together with recombinases, while IS6 bracketed the *mecA* gene and its regulatory genes (*mecI* and *mecR*) in three MRSA isolates (Table 4). Most ARGs, including *erm(A)*, *ant(9)-Ia*, *dfrG*, and *tet(M)*, were associated with a recombinase and integrase. One isolate was found harboring the *dfrG* gene bracketed by ISL3 and recombinases (Table 4).

#### Regulatory Genes

The accessory gene regulator system (*agr*) involved in the regulation and expression of toxins, exoenzymes, and biofilm was detected in all isolates. Isolates carried *agr* type I and II. The distribution of the *agr* group in MRSA was: *agr* I (n = 1), *agr* II (n = 2), while in MSSA *agr* I (n = 2), and *agr* II (n = 1).

### 2.7. Phylogenomics

**Figure 1:**
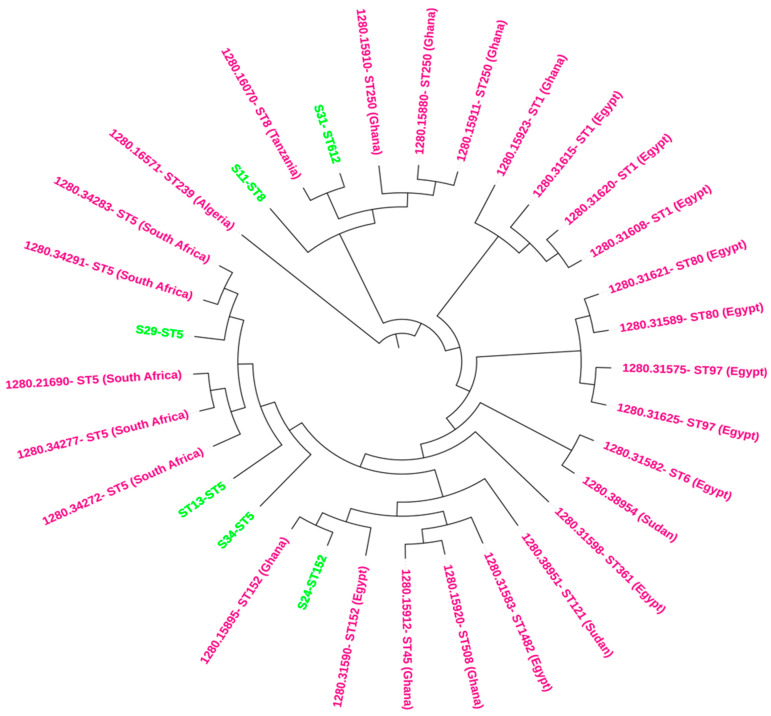
The circular phylogenetic tree provides a visual representation of the genetic relationships between *Staphylococcus aureus* isolates from this study (highlighted in green) and various African blood culture isolates (highlighted in purple). This tree illustrates how isolates cluster based on their Multilocus Sequence Typing (MLST) and geographic origin.

The phylogenetic analysis, integrated with metadata, reveals clear clustering patterns based on Multilocus Sequence Typing (MLST) and geographic origin. Isolates sharing the same MLST type generally clustered together, with additional grouping observed by country of isolation (Figure 1). For instance, the ST5 isolates from this study (S11-ST5, S34-ST5, and S29-ST5) are closely aligned with other South African isolates, indicating minimal genetic divergence within this MLST type in the region. This suggests a strong regional lineage for ST5 in South Africa. Additionally, this study isolates S24-ST152 clusters with other ST152 isolates from various African countries, including Kenya and Ghana, highlighting the broader geographic distribution and potential mobility of this MLST type across the continent (Figure 1). Notably, the tree analysis also reveals that ST8 and ST612 isolates are clustered together, suggesting close genetic relatedness despite being distinct MLST types (Figure 1). This finding could indicate a shared ancestry or recent genetic exchange between these groups, pointing to complex evolutionary dynamics within these populations.

**Figure 2:**
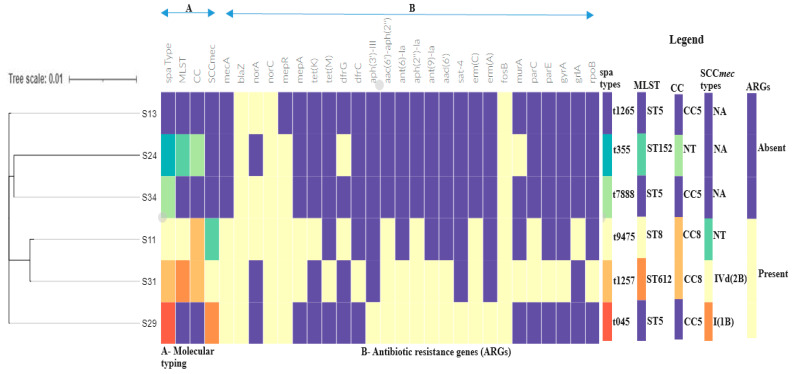
The phylogenetic branch and metadata, A—Molecular typing [*spa* type, sequence type (ST), clonal complex, SCC*mec* types], and B—Antibiotic resistance genes [ARGs], visualized using Phandango (https://github.com/jameshadfield/phandango/wiki accessed on 15 August 2024) in *S. aureus* isolates. The heat map in the middle indicates the presence (yellow) and absence (purple) of antibiotic resistance genes. NT indicates non-typeable. Isolates S13, S24, and S34 are MSSA and therefore do not harbor SCC*mec* types, which is indicated as “None (NA)” to reflect their SCC*mec*-negative status.

The linkage between the *mecA* gene, SCC*mec* types, and clonal complexes is particularly notable, as it highlights the genetic mechanisms underlying methicillin resistance and the clustering of MRSA strains (Figure 2). The distinct clustering patterns observed for different isolates underscore the complex interplay between genetic background, resistance gene acquisition, and selective pressures in the evolution of these pathogens.

**Table 3:** Genetic context of virulence genes in *S. aureus* isolates.

Strain (MLST)	Strain	Contig	Synteny of Virulence Genes and MGEs	Plasmid/Chromosomal Sequence with Closest Nucleotide Homology (Accession Number)
S11 (ST8)	MRSA	4	pmtC:pmtB:pmtA:***eap***::***scn***::***sak***:::sph::***lukG***::***lukH***::intergrase:::agrB	*S. aureus* strain Laus385 chromosome (CP071350.1)
		6	***icaR***::***icaD***:***icaB***:***icaC***:vraD:vraE:vraH::IS30:vraH::recombinase:IS6	*S. aureus* strain TF3198 chromosome, complete genome (CP023561.1)
		10	***lukE:lukD***::splA::epiE::***splA***:splB:splC:splD:splE:splF::pepA1:transposase	*S. aureus* strain 82 chromosome, complete genome (CP031661.1)
S29 (ST5)	MRSA	53	type I toxin–antitoxin system:IS6:cadD	*S. aureus* strain MIN-175 chromosome (CP086121.1)
		40	***clfA***:vwb:***emp***	*S. aureus* strain ER02693.3 chromosome, complete genome (CP030605.1)
S31 (ST612)	MRSA	11	pmtD:pmtC:pmtB:pmtA::***eap***:***scn***::***sak***	*S. aureus* strain 2395 USA500, complete genome (CP007499.1)
		15	***lukE*:*lukD***::::***splA***:***splB***:***splC***:***splF***::type I restriction-modification system	*S. aureus* strain NRL 02/947 chromosome, complete genome (CP103850.1)
		19	***lukG***:***lukH***:pathogenicity island:intergrase::phenol-soluble modulin:agrB	*S. aureus* strain 2395 USA500, complete genome (CP007499.1)
		22	***seq****:**sek***:integrase::::***emp***:***clfA***	*S. aureus* strain 2395 USA500, complete genome (CP007499.1)
		33	recombinase::universal stress protein:::cadD::***seq***:***sek***:integrase:::***emp***:***clfA***	*S. aureus* plasmid SAP017A, complete sequence (GQ900382.1)
		64	***sea***:putative holin-like toxin	*S. aureus* strain R50 chromosome, complete genome (CP039167.1)
S13 (ST5)	MSSA	4	***sbi***:***hlgA***:***hlgC***:***hlgB***	*S. aureus* strain AR462 chromosome, complete genome (CP029086.1)
		5	scpA:::**eap**::***scn***:***sak***::::::intergrase:sph:***lukH***:***sbi***:***hlgA***:***hlgC***:***hlgB***	*S. aureus* strain pt239 chromosome, complete genome (CP049467.1)
		15	IS6::cadD:::***sed***:***sej***:***ser***::recombinases:cpA::eap::***scn*:sak**::integrase:sph:***lukH***	*S. aureus* strain ER10678.3 plasmid pER10678.3A.1 (CP051928.1)
S24 (ST152)	MSSA	8	arsB::crcB::***scn***:***sak***:::recombinase::type II toxin–antitoxin system toxin:intergrase	*S. aureus* strain UMCG579 chromosome, complete genome (CP091066.1)
		21	cadD:type toxin–antitoxin::integrase	*S. aureus* strain GHA13 chromosome (CP043911.1)
		11	BrxA/BrxB:::msrA:msrB:::norD::cspA:cvfB	*S. aureus* strain NGA84b chromosome, complete genome (CP051165.2)
S34 (ST5)	MSSA	7	***eap/map***::***scn***:***sak***::::***sea***:::type II toxin–antitoxin:integrase:sph:***lukG***:***lukH***	*S. aureus* strain HPV107 chromosome, complete genome (CP026074.1)
		8	clfA:vwb:emp::thermonuclease protein:::s**ek**:***seq***::pathogenicity island	*S. aureus* strain B4-59C chromosome, complete genome (CP042153.1)
		12	**sem**:**sei**:**seu**:**sen**:**seg**:::***lukE***:***lukD***::splA:splB:splC:splD:splF	*S. aureus* strain ER03588.3 chromosome, complete genome (CP030595.1)
		14	isdB:isdA:isdC:isdD:isdE:isdF::isdG::***ecb****::**efb**:**scb***	*S. aureus* strain B3-17D chromosome, complete genome (CP042157.1)
		20	SSL13:SSL12:**hyl**	*S. aureus* strain NAS_AN_239 chromosome, complete genome (CP062409.1)

Virulence gene(s) in bold.

**Table 4:** Genetic environment of antibiotic resistance genes in *S. aureus* isolates.

Isolate ID (MLST)	Strain	Contig	Synteny of Resistance Genes and MGEs	Plasmid/Chromosomal Sequence with Closest Nucleotide Homology (Accession Number)
S11 (ST8)	MRSA	4	*blaI:blaR1:blaZ*::recombinase/integrase	*S. aureus* strain ER02826.3 chromosome (CP030661.1)
		7	recombinase::dfrG:insertionelement:::ISL3:::recombinase:	*S. aureus* strain UP_403 chromosome (CP047849.1)
		59	IS6::*mecA:MecR1*:IS6::	*S. aureus* strain ER03868.3 chromosome (CP030403.1)
		123	Plasmid recombination:*tet(K)*	*S. epidermidis* isolate BPH0662 genome assembly, plasmid: 1 (LT614820.1)
S29 (ST5)	MRSA	8	*erm(A):ant(9)-Ia*:transposase:recombinase:integrase	*S. aureus* strain 628 chromosome (CP022905.1)
		11	*gyrB:gyrA*:::ligase	*S. aureus* strain MIN-175 chromosome (CP086121.1)
		38	recombinase:IS1182::*mecR1:mecA*:::IS6	*S. aureus* subsp. aureus strain FDAARGOS_5 chromosome (CP007539.3)
		51 *	recombinase:*blaI:blaR1:blaZ:cadC:cadA*	*S. aureus* plasmid pSK57, partial sequence (GQ900493.1)
		56	*ant(6)-Ia:sat4:aph(3′)-IIIa*	*S. pseudintermedius* strain MAD627 chromosome (CP039743.1)
		64	*qacA/B*:qacR	*S. aureus* strain MIN-175 chromosome (CP086121.1)
		67	*ermCL:erm(C)*	*S. epidermidis* strain TMDU-137 plasmid p5, complete sequence (CP093178.1)
S31 (ST612)	MRSA	23	*mecA:mecR1*::IS1182::recombinase	*S. aureus* strain 2395 USA500 (CP007499.1)
		17	integrase::::::tet(M):::IS256	*S. aureus* strain NRS120 chromosome, complete genome (CP026072.1)
S13 (ST5)	MSSA	15 *	IS6 IS6::*cadD*:::type I toxin–antitoxin::recombinase::*blaI:blaR1:blaZ*	*S. aureus* strain ER10678.3 plasmid pER10678.3A.1 (CP051928.1)
S24 (ST152)	MSSA	21	cadD:typetoxinantitoxin::recombinase:*blaI*:*blaR1*:*blaZ*:recombinase	*S. aureus* strain GHA13 chromosome (CP043911.1)
S34 (ST5)	MSSA	19	recombinase:blaZ:blaR1:blaI:recombinase:integrase	*S. aureus* strain UP_678 plasmid unnamed (CP047840.1)

* Co-occurrence of a heavy metal resistance gene (HMRG) and antibiotic resistance genes (ARGs).

## 3. Discussion

We studied the genomic characteristics of six MDR *S. aureus* isolates implicated in BSIs. This study analyzed the resistome, virulome, mobilome, phylogeny, and genetic environment of the resistance genes using WGS and bioinformatics. The genomes analyzed herein were predominantly recovered from ≤1-year-old patients.

There was a diversity of ARGs encoding resistance to different antibiotics and good concordance between the observed phenotypic and genotypic resistance. The incidence of ARG’s encoding resistance to β-lactams, aminoglycosides, macrolides, fosfomycin, trimethoprim, tetracycline, and genes coding multidrug resistance (MDR) efflux pumps (*norA*, *mepR*, and *mgrA*) was not dependent on the clonal type. The *erm(C)* and *erm(A)* genes that are commonly found in macrolide–lincosamide–streptogramin B (MLS_B_)-resistant *S. aureus* were found in erythromycin and clindamycin-resistant isolates (Table 2), which was expected since resistance to erythromycin co-selects resistance to other antibiotics, such as streptogramin B (MLS_B_) and lincosamides [20]. The *ermC* gene is among the primary *erm* types that facilitate ribosome methylation of the 23S rRNA, triggering conformational changes resulting in drug binding inhibition [21], and has been reported in clinical *S. aureus* isolates from South Africa [22]. In this study, the *ermC* encoding macrolide resistance was carried on a plasmid, on a contig that had the closest nucleotide homology to plasmids from *S. epidermidis* strain TMDU-137 plasmid p5, complete sequence (CP093178.1), implying the likelihood of horizontal transfer of *ermC* genes in clinical *S. aureus* isolates. The *ermC* are often plasmid-mediated, resulting in high resistance to macrolides in *S. aureus* [23].

The *blaZ* gene, which inactivates penicillin through hydrolysis of the beta-lactam ring, was observed in all six isolates that were phenotypically resistant to penicillin. The *blaZ* genes have also been isolated in clinical isolates of Staphylococci in South Africa [24]. In this study, the *blaZ* genes were found on contigs with closest homology to either chromosomes or plasmids. This agrees with a study conducted in Spain that analyzed ARGs presence in chromosomes and plasmids from the genomes of *S. aureus*. WGS analysis of *S. aureus* revealed that *blaZ* (n = 2) was located on chromosomic contigs, while *blaZ* was found in plasmid contigs in three isolates [25]. It is important to note that most *blaZ* and associated MGEs from isolates belonging to ST5 (S13, S34) isolated from the intensive care unit (ICU) and pediatric ward (S29) were located on contigs that had the closest homology to plasmids, implying that plasmids play a crucial role in mobilizing the *blaZ* gene in clinical *S. aureus* isolates. The S29 isolate, belonging to the t045-CC5 lineage, carried an assortment of ARGs encoding resistance to different antibiotics (Table 4). Similar ARGs in MRSA lineage t045-CC5-MRSA were also reported in a study conducted in South Africa, where t045-CC5 MRSA lineages obtained from different clinical samples from South Africa and Nigeria reported that t045 lineages were MDR, suggesting that this lineage is hospital-associated and their multidrug resistance nature may compromise treatment [26].

Also, the *blaZ* genes, heavy metal genes, and associated MGEs were carried on either plasmid or chromosome. The *blaZ* and *cadAC* genes were found on the genetic element recombinase *blaI*:*blaR1*:*blaZ*:*cadC*:*cadA* for isolates S24 (MSSA) that was from the ICU and S29 (MRSA) from the pediatric ward, suggesting co-selection of heavy metal resistance dissemination and adaptation in different wards. The *cadA* gene confers a high resistance to cadmium and other heavy metals like zinc and lead in *S. aureus* isolates [27]. The *cadA* was associated with a plasmid, similar to the findings of a study that was conducted by Al-Trad et al. (2023) in Malaysia, who used WGS to analyze the plasmid content of clinical MRSA isolates and reported that heavy metal resistance plasmids harbored cadmium resistance genes, with the majority being *cadAC* [28]. The HMRGs have been reported to trigger a co-selection mechanism with antibiotics, which may complicate treatment [29]. This may pose a challenge, especially among patients in the ICU, where broad-spectrum antibiotics are often used.

Tetracycline resistance genes (*tetK* and *tetM*) were observed in two isolates. Isolate S11 carried *tet(K)* associated with the following genetic context: plasmid recombination *tet(K)* that had a high similarity to *S. epidermidis* BPH0662, and plasmid 1 (LT614820.1), which could be significant in mobilizing TET-resistant genes. Also, the *tet(M)* was bracketed by integrase and IS256 in isolate S31. The IS*256* is a retrotransposon that can mobilize the resistance genes through a copy-and-paste mechanism and has been shown to confer a robust genomic plasticity in MRSA strains [30].

We found that ARGs and virulence genes were associated with MGEs, which may enable their transfer within and between plasmids and chromosomes [31]. In this study, the *mecA* gene was located on IS1182 in two MRSA isolates, surrounded by recombinase in the genetic context *mecA:mecR1*::IS1182::recombinase. The insertion sequence IS1182 was present in 2/3 MRSA strains that contained *mecA*. IS1182 has been shown to occur close to the SCC*mec* element and increase resistance through inactivating the *lytH* gene encoding a putative lytic enzyme in pathogenic MRSA isolates [32].

MLST, clonal complex typing, *spa* typing, and SCC*mec* typing were used to analyze the molecular characteristics of the *S. aureus* isolates. Four ST types and two clonal clusters (CCs) were found among the six clinical isolates in this study, with ST5 as the most predominant complex clonal CC5 and CC8. Generally, clonal lineages ST5, ST8, ST152, and ST612 are among the most commonly reported in hospital environments, along with other sequence types of *S. aureus* [33]. *S. aureus* ST5, belonging to CC5, was predominant in this study and was previously reported among patients with bloodstream infections at Ruijin Hospital in Shanghai [3]. The detection of clonal complexes CC5 and CC8 agrees with a study by Smith et al. [17], which also found CC8 and CC5 were predominant in a study that analyzed the genomic epidemiology of MRSA and MSSA from bloodstream infections in the USA. Their results revealed that the MDR phenotype observed in strains belonging to CC5 and CC8 was responsible for the occurrence of multidrug and methicillin resistance in the *S. aureus* population. MRSA strains belonging to CC8 and CC5 are frequently associated with global outbreaks and have been identified in Africa [34].

The *spa* typing revealed six different *spa* types, suggesting a non-clonal MRSA and MSSA distribution. The detection of *spa* types t1257, t045, and t355 agrees with a study conducted in South Africa, which analyzed the diversity of SCC*mec* elements and *spa* types in *S. aureus* isolates from blood culture in the Gauteng, KwaZulu-Natal, Free State, and Western Cape provinces [15], in which t037 and t1257 were the most common and predominated throughout the seven-year study period. In this study, some antibiotic resistance genes were associated with specific MRSA clones belonging to *spa* types t1257, t045, and t9475. Shittu et al. (2021) found the *spa* types t045 and t1257 to be the most prevalent and associated with genes conferring resistance to aminoglycosides, trimethoprim, macrolides, and tetracycline in clinical isolates of *S. aureus* from South Africa and Nigeria [26].

The analysis of SCC*mec* types revealed the presence of SCC*mec* type IVd (2B) and SCC*mec* type I (B) carrying the *mecA* gene, which occurred in tandem with *mecR1* in both MRSA isolates. However, one MRSA (S11) isolate had a non-typeable SCC*mec* element cassette due to the missing cassette chromosome recombinase (*ccr*) gene complex [35]. The *ccr* gene complex is an essential component required to facilitate the integration or excision of the SCC*mec* element in the staphylococcal chromosome, and their loss has also been reported [36]. The SCC*mec* IV detected in our study is associated with the *spa* type t1257, previously reported in South Africa in *S. aureus* obtained from poultry isolates [16], implying its possible transfer between humans and animals.

We found different MRSA genotypes, ST612-t1257-CC8, ST8-t9475-CC8, and ST5-t045-CC5, suggesting that MRSA isolates were not clonally and epidemiologically related. The ST612-t1257-CC8 identified in this study is an endemic MRSA clone that has been reported in animal and clinical settings [15,16]. The ST5-I-MRSA, known as the pandemic British EMRSA-3 clone, was detected in the pediatric ward. This is similar to a study conducted in South Africa, where the t045-MRSA strain occurred in pediatric patients [19]. The isolation of the t045-ST5-MRSA strain could confirm its successful persistence in the hospital and its capacity to cause infections in neonatal and pediatric wards [37].

Several virulence factors, including adherence, immune invasion, toxins, and exoenzymes associated with invasive infections, were detected in our isolates. The virulence genes encoding clumping factor proteins (*clfA* and *clfB*) are involved in the pathogenesis of *S. aureus*, including bacteremia [9]. Consistent with pathogenic *S. aureus* strains isolated in various environments globally, our isolates were characterized by the *icaADBC* operon and *sdrC*, *sdrD*, and *sdrE* involved in biofilm-forming genes [38]. Most strains harbored genes, including the alpha and gamma-hemolysin genes (*hlgA*, *hlgB*, *hlgC*, *hly/hla*, and *hlb*), and the *ica* operon associated with pathogenicity and adhesion. Additionally, our isolates were characterized by various toxins, including *lukE/D* genes and Panton–Valentine leukocidin (PVL) *lukS-PV*/*lukF-PV* genes in one MSSA and MRSA strain. The expression of these PVL toxin genes in *S. aureus* isolates lyses host cells and promotes virulence of the bacteria [39], which might worsen the outcomes of *S. aureus* infection. Consistent with clinical *S. aureus* strains, our isolates were characterized by a capsular polysaccharide (CP) serotype 8, which shields the bacterial pathogen from host immune defense mechanisms associated with increased virulence in BSIs [40].

Most virulence genes, including those encoding SEs, *sak*, *hlg*, *luk*, *scn*, *clfA*, *sbi*, and associated MGEs, were carried on chromosomes in the majority of isolates. The *ica* gene operon and *vra* genes were found to be associated with ISs (IS30, IS6) and recombinase for S11 (ST8) isolate from the surgical ward. The *ica* genes *vraDEH* genes have been shown to play an important role in biofilm formation [41] and daptomycin resistance in *S. aureus* [42], which could enhance antibiotic resistance traits and chronic infection. The occurrence of ST8-t9475 MRSA strains co-harboring *ica* genes and genes encoding daptomycin resistance in ST8 MRSA could be advantageous to the ST8-t9475 colonization, invasion, and survival in the surgical ward. The virulence genes encoding SEs, *eap*, *scn*, *sak*, *sph*, *lukH*, and *cadA*, were found on a contig that had high sequence similarity to *S. aureus* strain ER10678.3 plasmid pER10678.3A.1 (CP051928.1), implying that they are mobilized by plasmids. Virulence genes, including those encoding *hla*/*hld*, toxin production, and biofilm formation, are plasmid-mediated [43], thus could easily facilitate their transfer, resulting in highly pathogenic strains that may be difficult to treat.

Phylogenomic analyses revealed that the clinical isolates in this study clustered mainly with clinical isolates from hospital patients (Figure 2). ST5 study isolates were closely related to clinical isolates from South Africa, suggesting possible dissemination of ST5 strains and adaptation in hospital environments. Furthermore, the ST152 isolate was closely related to ST152 strains from Egypt and Ghana, implying a possible spread and epidemiological linkage between these isolates. ST152-PVL-producing *S. aureus* isolates are particularly frequent and widespread in West and Central Africa [44] and livestock [45]. The ST152-PVL-positive MSSA has also been reported from cutaneous abscesses among mine workers at a gold mine in Gauteng, South Africa [46]. Identifying ST152 in livestock and humans suggests animal–human transmission, which requires further investigation. ST8 and ST612 isolates were closely related to ST8 isolated from Tanzania, indicating that ST612 is a double-locus variant of ST8. ST8 and ST612 isolates are potentially multidrug-resistant and highly virulent strains associated with hospital outbreaks [47]. The integration of phylogenetic data with resistance profiles offers valuable insights into the epidemiology and evolutionary dynamics of these isolates, highlighting potential patterns of transmission and resistance development [48].

In light of the increasing resistance observed in *S. aureus* strains, innovative strategies are being explored to combat antimicrobial resistance. One promising approach focuses on targeting microbial metallophores, molecules that bacteria use to scavenge essential metals from their environment. By inhibiting metallophore function, it is possible to disrupt bacterial metabolism and enhance the effectiveness of existing antibiotics [49]. Additionally, alternative strategies, such as the use of bacteriophages, antimicrobial peptides, and immune system modulation, offer potential avenues for treating resistant infections [50]. Finally, combination therapy, which involves using multiple antibiotics or combining antibiotics with adjuvants that inhibit resistance mechanisms, is gaining attention as a way to overcome multi-drug resistance and reduce the likelihood of treatment failure [51]. These emerging strategies represent critical avenues for future research and clinical application, aiming to curb the growing threat of antimicrobial resistance.

This study analyzed a limited number of *Staphylococcus aureus* isolates (n = 6), which may not fully represent the epidemiology of MRSA/MSSA in South Africa or bloodstream infections more broadly. The small sample size limits the generalizability of the findings to the wider population. Therefore, while this study offers valuable insights into the genomic characteristics and resistance profiles of these isolates, it should be viewed as a pilot study that lays the groundwork for larger, more comprehensive investigations. The small sample size also limits the ability to validate the findings, particularly concerning virulence factors and other genomic features. Future studies with larger sample sizes and experimental validation are necessary to confirm and extend these observations.

## 4. Materials and Methods

### 4.1. Ethical Consideration

The ethical approval for this study was issued by the Biomedical Research Ethics Committee of the University of KwaZulu-Natal under the following reference number: BCA444/16.

### 4.2. Sample Collection and Bacterial Identification

A total of forty-five presumptive *Staphylococcus* isolates from blood cultures sourced from patients with BSIs at two hospitals in the uMgungundlovu district in the KwaZulu-Natal province from November 2017 to December 2018. All isolates were confirmed as *S. aureus* using the automated VITEK 2 system (BioMérieux, MarcyL’Etoile, France). We selected a subset of 10 MDR isolates for WGS based on their antibiotic-resistant profiles/patterns, but 4 isolates were excluded during the quality control process.

### 4.3. Antimicrobial Susceptibility and MRSA Detection

Isolates were tested for antibiotic susceptibility by disk-diffusion method on Mueller–Hinton agar as recommended by the European Committee on Antimicrobial Susceptibility Testing (EUCAST) [52] or Clinical and Laboratory Standards Institute (CLSI) [53]. The antibiotics tested and interpreted according to the EUCAST breakpoints (EUCAST, 2017) were penicillin G (10 µg), ampicillin (10 µg), cefoxitin (30 µg), tigecycline (15 µg), and nitrofurantoin (300 µg). The CLSI guidelines (CLSI, 2017) were used for the following antibiotics: ciprofloxacin (5 µg), levofloxacin (5 µg), moxifloxacin (5 µg), erythromycin (15 µg), gentamicin (10 µg), amikacin (30 µg), chloramphenicol (30 µg), tetracycline (30 µg), doxycycline (30 µg), sulphamethoxazole/trimethoprim (1.25 µg + 23.75 µg), teicoplanin (30 µg), linezolid (30 µg), clindamycin (2 µg), and rifampicin (5 µg). MRSA isolates were identified using a cefoxitin disk (30 μg). The antibiotic disks were obtained from Oxoid (Oxoid, Basingstoke, UK). *S. aureus* ATCC 29213, was used as the quality control strain. Multidrug resistance (MDR) was defined as resistance to three or more antibiotic classes [54].

### 4.4. Whole-Genome Sequencing (WGS) and Bioinformatic Analysis

Genomic DNA extraction was performed using the GenElute Bacterial Genomic DNA kit (Sigma Aldrich, St. Louis, MO, USA) following the manufacturer’s instructions. The quality of the DNA was assessed using NanoDrop 8000 (Thermo Fisher Scientific Waltham, MA, USA). Genome libraries were constructed using the Nextera XT DNA Library Preparation Kit (Illumina, San Diego, CA, USA) and sequenced on the Illumina NextSeq Machine (Illumina, San Diego, CA, USA). The raw reads were trimmed using Sickle v1.33 (https://github.com/najoshi/sickle accessed on 15 August 2020) and assembled using the SPAdes v3.6.2 assembler (https://cab.spbu.ru/software/spades/ accessed on 15 August 2020). Assembled genome sequences were submitted to Genebank and assigned accession numbers under the BioProject number PRJNA400143.

### 4.5. Genomic Analysis

Genotyping of the assembled genomes was performed using the MLST 2.0, *spa* Typer 1.0, and SCC*mec*Finder 1.2 available at the Centre for Genomic Epidemiology (CGE) (https://www.genomicepidemiology.org/services/ accessed on 1 October 2023). The detection of antibiotic resistance genes (ARGs) was examined by ResFinder 4.1 (https://cge.cbs.dtu.dk/servic es/ResFinder/ accessed on 1 October 2023) and the comprehensive antibiotic resistance database (https://card.mcmaster.ca/analyze/rgi accessed on 1 October 2023). Virulence determinants were identified with default settings using the virulence factor database (VFDB: http://www.mgc.ac.cn/VFs/main.htm accessed on 15 December 2023) and VirulenceFinder 2.0 https://cge.food.dtu.dk/services/VirulenceFinder/ accessed on 1 October 2023). Pathogenicity of isolates was found out using PathogenFinder 1.1 (https://cge.food.dtu.dk/services/PathogenFinder/ accessed on 1 October 2023). PHASTER was used to identify prophage elements (https://phaster.ca/ accessed on 1 October 2023). Mobile genetic elements (MGEs) in relation to ARGs, virulent factors, and plasmid replicons were identified using MobileElementFinder (https://cge.food.dtu.dk/services/MobileElementFinder/ accessed on 1 October 2023) and Plasmid Finder 2.1 (https://cge.cbs.dtu.dk/services/PlasmidFinder/ accessed on 1 October 2023). The genetic environment of ARGs, virulence factors, and associated MGEs was examined using GenBank’s general feature format (GFF3) files and imported into Geneious Prime 2020.2 (https://www.geneious.com accessed on 10 December 2023) for analysis [55]. The accessory gene regulator (*agr*) typing was performed by employing nucleotide BLAST, and the following GenBank accession numbers: AFS50129.1, AFS50128.1, AFS50130.1, and AFS50131.1 were used as reference sequences for *agr* types I–IV [56].

### 4.6. Phylogenomic Analysis

For phylogeny analysis, whole-genome sequences of *S. aureus* isolates from blood culture were selected from Northern Africa (Egypt, Algeria, and Sudan), Western Africa (Ghana), and Eastern Africa (Tanzania) and downloaded from the bacterial and viral bioinformatics resource center’s (BV-BRC) online platform (https://www.bv-brc.org/ accessed on 15 May 2024) and used together with our study’s isolates. We constructed a phylogenomic tree using the online Phylogenetic Tree Building tool available on the BV-BRC website (https://www.bv-brc.org/ accessed on 15 May 2024). The generated phylogenetic tree was visualized, annotated, and edited using iTOL (https://itol.embl.de/ accessed on 15 May 2024) and Figtree (http://tree.bio.ed.ac.uk/software/figtree/ accessed on 15 May 2024).

### 4.7. Nucleotide Sequence Accession Number

The nucleotide sequences of MRSA (S29, S11, S31) and MSSA (S13, S24, S34) isolates were submitted to the NCBI GenBank database under the following accession numbers: JADQTH000000000, JADIXB000000000, JADIXC000000000, JADIXA000000000, JADIXE000000000, and JADIXD000000000.

## 5. Conclusions

This study presents an insight into ARGs, virulence genes, MGEs, and genetic diversity of *S. aureus* collected from a public hospital in uMgungundlovu. We observed high diversity of *spa* types, STs, and a predominance of CC8 and CC5, indicating the genetic variability of *S. aureus* in hospital settings. The occurrence of pathogenic and MDR strains in the hospital setting, especially in the ICU, can pose a serious threat that limits the therapeutic options available. Here, we demonstrate that while MRSA displayed multidrug resistance, MSSA reflected potentially increasing resistance to the antibiotics used for treatment. Continuous surveillance and monitoring of MRSA and MSSA strains circulating in hospital environments is needed.

## Figures and Tables

**Table 1:** Antibiotic susceptibility profiles, age, and demographic characteristics of patients with BSIs attributed to *S. aureus*.

Isolate ID	Species	Sex	Ward	Age	Antibiotics																			
					PEN	AMP	FOX	CIP	MXF	LEV	GEN	AMK	ERY	CLI	TET	DOX	TGC	CHL	NIT	SXT	VAN	RIF	LZD	TEC
S11	MRSA	F	Surgical ward	17 years	R	R	R	R	R	R	R	R	R	R	R	R	R	I	S	R	S	R	R	R
S29	MRSA	M	Pediatric ward	<1 year	R	R	R	R	R	R	R	R	I	R	R	R	S	I	S	R	S	I	R	R
S31	MRSA	F	Surgical ward	3 years	R	R	R	R	R	R	R	R	R	R	R	R	S	R	S	R	S	R	R	R
S24	MSSA	M	ICU	33 years	R	S	S	R	R	R	S	S	R	R	R	I	S	I	R	R	S	R	I	I
S13	MSSA	M	ICU	<1 year	R	S	S	R	R	R	I	R	I	R	I	R	S	S	S	I	S	S	I	R
S34	MSSA	M	NICU	<1 year	R	S	S	R	R	R	I	R	I	R	R	R	S	I	S	S	S	I	S	S

Key: PEN, penicillin; AMP, ampicillin; FOX, cefoxitin; CIP, ciprofloxacin; MXF, moxifloxacin; LEV, levofloxacin; GEN, gentamicin; AMK, amikacin; ERY, erythromycin; CLI, clindamycin; TET, tetracycline; DOX, doxycycline; TGC, tigecycline; CHL, chloramphenicol; NIT, nitrofurantoin; SXT, trimethoprim-sulfamethoxazole; VAN, vancomycin; RIF, rifampicin; LZD, linezolid; TEC, teicoplanin. R, resistant; I, intermediate; S, susceptible; M, male; F, female; NICU, neonatal intensive care unit; ICU, intensive care unit.

**Table 2:** Genotypic characteristics of *S. aureus* implicated in BSIs.

Isolate ID	MRSA/MSSA	MLST	*spa* Type	Resistome	Plasmid Replicon Type	Insertion Sequences	Confirmed CRISPRs (CAS)	Clonal Complex	* SCC*mec* Type	*agr* Type	Pathogenicity Score
S11	MRSA	ST8	t9475	*blaZ*, *mecA*, *aac(6′)-aph(2*″*)*, *parC*, *dfrG*, *erm(C)*, *grlA*, *tetK*, *mepR*, *mepA*, *norA*, *norC*, *fosB*	rep10, rep7a, rep7c	-	6 (0)	CC8	NT	Type I	0.982 (882)
S29	MRSA	ST5	t045	*blaZ*, *mecA*, *aph(3′)-III*, *aac(6′)-aph(2*″*)*, *ant(6)-Ia*, *ant(9)-Ia*, *aad(6′)*, *erm(C)*, *erm(A)*, *qacA*, *mepR*, *fosB*, *norA*, *norC*, *sat-4*	rep10, rep21	IS6, IS256	12 (0)	CC5	SCC*mec* type I(1B)	Type II	0.98 (914)
S31	MRSA	ST612	t1257	*blaZ*, *mecA*, *aac(6′)-aph(2*″*)*, *aph(2*″*)-Ia*, *aad(6′)*, *ant(6)-Ia*, *ant(9)-Ia*, *tet(M)*, *mepR*, *mepA*, *dfrC*, *parC*, *erm(C)*, *parE*, *gyrA*, *rpoB*, *fosB*, *norA*, *norC*, *murA*	rep7c, rep20	IS256, IS6	7 (0)	CC8	SCC*mec* type IVd(2B)	Type I	0.976 (978)
S24	MSSA	ST152	t355	*blaZ*, *dfrG*, *mepR*, *norC*, *murA*	rep16, rep5a	-	8 (0)	-	NA	Type IV	0.975(225)
S13	MSSA	ST5	t1265	*blaZ*, *norA*, *norC*, *fosB*	rep20	-	9 (0)	CC5	NA	Type II	0.985 (844)
S34	MSSA	ST5	t7888	*blaZ*, *norA*, *norC*, *mepR*, *fosB*	rep19, rep16, rep20, rep5a	1S6	7 (0)	CC5	NA	Type II	0.983 (871)

* SCC*mec* typing was predicted with the SCC*mec*Finder, MSSA—Methicillin-susceptible *Staphylococcus aureus*, MRSA—Methicillin-resistant *Staphylococcus aureus*—non-typeable (NT). The MSSA do not harbor SCC*mec* types, which is indicated as “None (NA)” to reflect their SCC*mec*-negative status.

## Data Availability

The data presented in this study are openly available in GenBank and assigned accession numbers under the BioProject PRJNA400143. [NCBI Genebank] [https://www.ncbi.nlm.nih.gov/bioproject/?term=PRJNA400143 accessed on 15 May 2024] [PRJNA400143].

## Supplementary Materials

The following supporting information can be downloaded at: https://www.mdpi.com/article/10.3390/antibiotics13090796/s1. Table S1: Patient demographics. Table S2: Genomic characteristics of *S. aureus* strains. Table S3: Virulence genes identified in MSSA and MRSA isolates in this study. Table S4: Distribution of insertion sequences and plasmid replicon among the *Staphylococcus aureus* strains. Table S5: Distribution of intact prophage region among the *Staphylococcus aureus* strains.
