# Supplementary material for: Genomic Characterization of Methicillin-Resistant and Methicillin-Susceptible Staphylococcus aureus Implicated in Bloodstream Infections, KwaZulu-Natal, South Africa: A Pilot Study
Source: Antibiotics (Basel). 2024 Aug 23;13(9):796. doi: 10.3390/antibiotics13090796 (PMC11429224; doi:10.3390/antibiotics13090796)
Supplement: Supplementary file 1 [file antibiotics-13-00796-s001.zip › antibiotics-3129108-supplementary.pdf]

antibiotics

Supplementary Materials

Genomic Characterization of Methicillin-Resistant and Methicillin-Susceptible *Staphylococcus aureus* Implicated in Bloodstream Infections, KwaZulu-Natal, South Africa: A Pilot Study

Bakoena A. Hetsa [1,*], Jonathan Asante [1,2], Joshua Mbanga [1,3], Arshad Ismail [4,5], Akebe L. K. Abia [1], Daniel G. Amoako [1,6] and Sabiha Y. Essack [1,7]

1 Antimicrobial Research Unit, College of Health Sciences, University of KwaZulu-Natal, 4000 Durban, South Africa; jonathan.asante@ucc.edu.gh (J.A.); mbangaj@ukzn.ac.za (J.M.); abiaakebel@ukzn.ac.za (A.L.K.A.); amoakod@ukzn.ac.za (D.G.A.); essacks@ukzn.ac.za (S.Y.E.)
2 School of Pharmacy and Pharmaceutical Sciences, University of Cape Coast, PMB, Cape Coast, Ghana
3 Department of Applied Biology & Biochemistry, National University of Science and Technology, Corner Cecil Avenue & Gwanda Road, Bulawayo 263, Zimbabwe
4 Sequencing Core Facility, National Institute for Communicable Diseases, Division of the National Health Laboratory Service, Johannesburg 2193, South Africa; arshadi@nicd.ac.za
5 Department of Biochemistry and Microbiology, Faculty of Science, Engineering and Agriculture, University of Venda, Thohoyandou 0950, South Africa
6 Department of Pathobiology, University of Guelph, Guelph, ON N1G 2W1, Canada
7 School of Pharmacy, University of Jordan, Amman 11942, Jordan
* Correspondence: 218085934@stu.ukzn.ac.za

Table S1. Patients demographics.

Isolate ID	Location/ Hospital	Department	Gender	Age
S11	Regional hospital	Surgical ward	F	17 years
S24	Regional hospital	ICU	M	33 years
S29	Regional hospital	Paediatric ward	M	<1 year
S13	Regional hospital	ICU	M	<1 year
S31	Regional hospital	Surgical ward	F	3 years
S34	Regional hospital	NICU	M	<1 year

Key: M, male; F, female; NICU, neonatal intensive care unit; ICU, intensive care unit.

Table S2. Genomic characteristics of *S. aureus* strains.

Isolate ID	Accession no	Size (Mb)	GC%	Contigs	No. of RNAs	No of coding Sequences	N50	L50	Coverage
S11	JADQTH000000000	2.7	32.7	37	65	2629	421,368	2	95.7
S24	JADIXC000000000	2.7	32.7	47	69	2571	330,697	4	95.7
S29	JADIXA000000000	2.8	32.8	95	69	2798	77,541	14	99.2
S13	JADIXB000000000	2.7	32.7	32	69	2550	695,668	2	95.7
S31	JADIXE000000000	2.9	32.7	106	69	2942	155,137	6	102.8
S34	JADIXD000000000	2.7	32.6	52	63	2635	273,350	5	95.7

Table S3. Virulence genes identified in MSSA and MRSA isolates in this study.

Isolate ID	Adherence factors	Immune envasion	Enzymes	Iron sequestration	Capsule biosynthesis	Toxins	Type VII secretion
S11	*atl, ebh, clfA, ebp, efb, fnbA, fnbB, icaA, icaB, icaC, icaR, sdrC, sdrE,* spa	*adsA, chp, scn, sbi*	*sspB, sspC, hysA, geh, lip, sspA, splA, splB, splC, splD, splE, splF, coa, sak, nuc,* aur	*isdA, isdB, isdC, isdD, isdE, isdF, isdG*	cap8A, cap8B, cap8C, cap8D, cap8E, cap8F, cap8G, cap8L, cap8M, cap8N, cap8O, cap8P	*hlgA, hlgB, hly/hla, hlb, hld, selk, eta, sek set21, set30, set31, set34, set36, set37, set38, set39, set40, set8, hlgA, hlgB, hlgC, lukD*	esaA, esaB, esaD, esaE, esaG, essA, essB, essC, esxA, esxB, esxC, esxD
S24	*atl, cna, ebp, fnbA, fnbB, icaA, icaB, icaC, icaR, sdrE, sdrD, spa*	*adsA, chp, scn, sbi*	*sspB, sspC, hysA, geh, lip, sspA, coa, sak, nuc,* aur	*isdA, isdB, isdC, isdD, isdE, isdF, isdG*	cap8A, cap8B, cap8C, cap8D, cap8E, cap8F, cap8G, cap8L, cap8M, cap8N, cap8O, cap8P	*hlgA, hlgB, hlgC, hly/hla, hlb, hld, eta, set15, set16, set18, set34, set36, set4, set7, lukD, lukE, lukF-PV, lukS-PV*	esaA, esaD, esaE, esaG, essA, essB, essC, esxA, esxB, esxC, esxD

S29	*atl, ebh, clfA, clfB, ebp, efb, fnbA, icaA, icaB, icaC, icaR, sdrC, sdrE, spa*	*adsA, sbi*	*sspB, sspC, hysA, geh, lip, sspA, splA, splB, splC, splD, splF, coa, nuc,* aur	*isdA, isdB, isdC, isdD, isdE, isdF, isdG*	cap8A, cap8B, cap8C, cap8D, cap8E, cap8F, cap8G, cap8L, cap8M, cap8N, cap8O, cap8P	*hlgA, hlgB, hlgC, hld, seg, yent1, yent2, selk, selm, seln, selo, eta, set11, set13, set15, set34, set37, set39, set6, set7, set8, set9, lukD, cylR2*	*esaA, esaD, esaE, esaG, essA, essB, essC, esxA, esxB, esxC, esxD*
S13	*atl, ebh, clfB, ebp, , efb, fnbA, fnbB, icaA, icaB, icaC, icaR, sdrC, spa*	*adsA, chp, scn, sbi*	*sspB, sspC, hysA, geh, lip, sspA, splA, splB, splC, splD, splF, coa, sak, nuc,* aur	*isdA, isdB, isdC, isdD, isdE, isdF, isdG*	cap8A, cap8B, cap8C, cap8D, cap8E, cap8F, cap8G, cap8L cap8M, cap8N, cap8O, cap8P	*hlgA, hlgB, hlgC, hly/hla, hlb, hld, sea, sej, sed, ser, seo, yent1, yent2, selk, selm, seln, selo, eta, set11, set13, set15, set34, set37, set39, set6, set7, set8, set9, lukE, lukD*	*esaA, esaB, esaD, esaE, esaG, essA, essB, essC, esxA, esxB, esxC, esxD*
S31	*atl, ebh, clfA, clfB, ebp, efb, fnbA, icaA, icaB, icaC, icaR, sdrC, sdrD, sdrE, sdrE, spa*	*adsA, chp, scn, sbi*	*sspB, sspC, hysA, geh, lip, sspA, splA, splC, splD, coa, sak, nuc,* aur	*isdA, isdB, isdC, isdD, isdE, isdF, isdG*	cap8A, cap8B, cap8C, cap8D, cap8E, cap8F, cap8G, cap8L cap8M, cap8N, cap8O, cap8P	*hlgA, hlgB, hlgC, hly/hla, hlb, hld, sea, seb, selk, selq, eta, set18, set21, set30, set31, set34, set36, set37, set38, set39, set40, lukD*	*esaA, esaB, esaD, esaE, esaG, essA, essB, essC, esxA, esxB, esxC, esxD*

S34	atl, clfA, clfB, ebp, efb, fnbA, fnbB, icaA, icaB, icaC, icaR, *sdrE*, *spa*	adsA, chp, scn, sbi	*sspB*, *sspC*, *hysA*, *geh*, *lip*, *sspA*, *splA*, *splC*, *splD*, *splF*, *coa*, *sak*, *nuc*, *aur*	*isdA*, *isdB*, *isdC*, *isdD*, *isdE*, *isdF*, *isdG*	cap8A, cap8B, cap8C, cap8D, cap8E, cap8F, cap8G, cap8L cap8M, cap8N, cap8O, cap8P	*hlgA*, *hlgB*, *hlgC*, *hly/hla*, *hlb*, *hld*, *sea*, *seb*, *seg*, *yent1*, *yent2*, *selk*, *selm*, *seln*, *selo*, *selp*, *selq*, *eta*, *set11*, *set13*, *set15*, *set34*, *set37*, *set39*, *set6*, *set7*, *set8*, *set9*, *lukD*	*esaA*, *esaB*, *esaD*, *esaE*, *esaG*, *essA*, *essB*, *essC*, *esxA*, *esxB*, *esxC*, *esxD*

Table S4. Distribution of insertion sequences and plasmid replicon among the *Staphylococcus aureus* strains.

Isolate	ST	Insertion sequence (IS)	Plasmid replicon
S11	ST8	-	Rep10
		-	Rep7a
		-	-
S13	ST5	-	Rep20
S24	ST152	-	Rep16
S29	ST5	-	Rep10
		IS6	-
		IS6	-
		IS256	-
S31	ST612	IS256	-
		IS256	-
		IS6	-
		-	Rep7c
		-	Rep20
S34	ST5	-	Rep19

Key: ST, sequence types; "-", no hits found.

Table S5. Distribution of intact prophage region among the *Staphylococcus aureus* strains.

Isolate ID	No. of prophage	Region	Length (Kb)	No. CDS	GC%	Phage (Hit genes count)	Resistance
S11	1	3	73.5	73 534	32.03	PHAGE_Staphy_P282	-
S13	1	2	45.2	45 286	33.01	PHAGE_Staphy_P282	-
S24	1	1	73.5	73 537	32.88	PHAGE_Staphy_phi2958PVL	-
S29	2	2	26.8	26 885	36.85	PHAGE_Staphy_SA13	-
		3	31.5	31 540	33.65	PHAGE_Staphy_phi2958PVL	-
S31	1	2	48.4	48 460	34.74	PHAGE_Staphy_phiJB	*dfrG*
S34	-	-	-	-	-	-	-

Key: "-", no hits found.
